# Sentinel lymph node mapping with indocyanine green in cervical cancer patients undergoing open radical hysterectomy: a single-institution series

**DOI:** 10.1007/s00432-020-03393-6

**Published:** 2020-09-30

**Authors:** Nicolò Bizzarri, Pedone Anchora Luigi, Gabriella Ferrandina, Gian Franco Zannoni, Maria Vittoria Carbone, Camilla Fedele, Elena Teodorico, Valerio Gallotta, Salvatore Gueli Alletti, Vito Chiantera, Anna Fagotti, Giovanni Scambia, Francesco Fanfani

**Affiliations:** 1 UOC Ginecologia Oncologica, Dipartimento per la salute della Donna e del Bambino e della Salute Pubblica, Fondazione Policlinico Universitario A. Gemelli, IRCCS, Largo Agostino Gemelli 8, 00168 Rome, Italy; 2 Università Cattolica del Sacro Cuore, Largo Francesco Vito 1, 00168 Rome, Italy; 3 Unità di Gineco-Patologia e Patologia Mammaria, Dipartimento per la salute della Donna e del Bambino e della Salute Pubblica, Fondazione Policlinico Universitario A. Gemelli, IRCCS, Largo Agostino Gemelli 8, 00168 Rome, Italy; 4 Department of Gynecologic Oncology, ARNAS Ospedali Civico di Cristina Benfratelli, University of Palermo, Piazza Nicola Leotta 4/A, 90127 Palermo, Italy

**Keywords:** Cervical cancer, Sentinel lymph node, Laparotomy, Minimally invasive surgery, Indocyanine green, Detection rate

## Abstract

**Purpose:**

To assess the rate of bilateral sentinel lymph node (SLN) detection with indocyanine green (ICG), to evaluate the sensitivity and the negative predictive value of cervical cancer patients undergoing open radical hysterectomy; to compare open versus minimally invasive SLN biopsy performance and to assess factors related to no/unilateral SLN mapping.

**Methods:**

We retrospectively reviewed consecutive patients with FIGO 2018 stage IA1 with lymph-vascular space involvement to IIB and IIIC1p cervical carcinoma who underwent SLN mapping with ICG followed by systematic pelvic lymphadenectomy between 05/2017 and 06/2020. Patients were divided according to surgical approach for statistical analysis.

**Results:**

Eighty-five patients met inclusion criteria. Twenty-seven (31.8%) underwent open and 58 (68.2%) underwent minimally invasive SLN mapping. No difference in any SLN mapping (laparotomy 92.6% and minimally invasive 91.4%) or in SLN bilateral detection (laparotomy 72.0% and minimally invasive 84.9%) (*p* = 0.850 and *p* = 0.222, respectively), in median number of SLNs mapped and retrieved (2 in both groups, *p* = 0.165) and in site of SLN mapping per hemi-pelvis (right side, *p* = 0273 and left side, *p* = 0.618) was evident between open and minimally invasive approach. Per-patient sensitivity of SLN biopsy in laparotomy was 83.3% (95% CI 35.9–99.6%) and the negative predictive value was 95.0% (95% CI 76.0–99.1%). No difference in per-patient sensitivity was noted between two approaches (*p* = 0.300). None of the analyzed variables was associated with no/unilateral SLN mapping.

**Conclusion:**

The use of ICG to detect SLN in cervical cancer treated with open surgery allows a bilateral detection, sensitivity and negative predictive value comparable to minimally invasive surgery with potential advantages of ICG compared to other tracers.

**Electronic supplementary material:**

The online version of this article (10.1007/s00432-020-03393-6) contains supplementary material, which is available to authorized users.

## Introduction

Despite the introduction of screening and vaccination has significantly reduced the incidence of cervical cancer in developed countries (Brisson 2020; Wright 2015), this tumor still represents the fourth most common cancer worldwide, with 569,847 new cases and 311,365 deaths in 2018 (Arbyn 2020). Treatment of early-stage disease (clinical International Federation of Gynecology and Obstetrics (FIGO) stage IA1-IB1 and IIA1) is represented by radical hysterectomy and pelvic lymphadenectomy with sentinel lymph node (SLN) biopsy (Cibula 2018). After the publication of a randomized controlled trial, which demonstrated an inferior survival outcome if patients with early-stage cervical cancer were treated with minimally invasive, compared to open radical hysterectomy (Ramirez 2018), multiple retrospective studies showed a survival difference between the two approaches, with consequent recommendations amendment by international societies (Koh 2019; Nitecki 2020; Querleu 2020), particularly for tumors larger than 2 cm (Pedone Anchora 2020).

SLN biopsy has been largely studied in cervical cancer and multiple evidences support its use in early-stage disease treated with primary surgery (Cibula 2018,2019); also, few studies reported the use of SLN biopsy after neo-adjuvant chemotherapy (NACT) (Slama 2012; Robova 2014). Different studies reported the adoption of SLN in open radical surgery with blue dye or radioactive tracer (Silva 2005; Wydra 2006; Altgassen 2007; Kara 2008; Dostalek 2020), but experience on the use of indocyanine green (ICG) in open surgery, is limited to few case reports or case series (Furukawa 2010; Crane 2011; van der Vorst 2011; Schaafsma 2012; Buda 2016; Rychlik 2016). On the other hand, it seems well established that ICG has been showed to be superior to blue dye or radioactive tracer in SLN bilateral detection rate in minimally invasive surgery (Di Martino 2017).

The primary objective of the present study was to assess the rate of bilateral SLN detection with ICG in cervical cancer patients undergoing open radical hysterectomy. Secondary objectives were to evaluate the sensitivity and the negative predictive value of open ICG SLN biopsy, to compare open versus minimally invasive SLN biopsy performance and to assess factors related to no/unilateral SLN mapping.

## Methods

### Inclusion and exclusion criteria

The present retrospective observational cohort study was approved by Institutional Review Board (Dipartimento per la salute della Donna e del Bambino e della Salute Pubblica, number DIPUSVSP-26-05-2059, date 26/05/2020). After signing consent form, consecutive patients who underwent surgery at Fondazione Policlinico A. Gemelli IRCCS (Rome, Italy), for International Federation of Gynecology and Obstetrics (FIGO) 2018 (Bhatla 2018) stage IA1 with lymph-vascular space involvement (LVSI) to IIB and IIIC1p cervical carcinoma between 05/2017 and 06/2020 and had SLN mapping attempt with systematic pelvic lymphadenectomy, were included. Data were retrieved from institution’s electronic database. All patients had pre-operative histology confirmation of cervical cancer and underwent pre-operative abdominal Magnetic Resonance Imaging (MRI) scan, pelvic ultrasound scan and chest X-ray. Patients with FIGO stage > IB1 underwent whole body positron emission tomography (PET)/computed tomography (CT) scan to exclude distant metastasis. Radicality of hysterectomy was classified according to Querleu-Morrow classification (Querleu 2017). Examination under anesthesia was performed at the same time of the surgery. Only women with no evidence of enlarged (i.e., short axis > 10 mm) pelvic and para-aortic lymph nodes at pre-operative MRI-scan were submitted to SLN mapping. Patients with pre-invasive cervical disease, who did not undergo systematic pelvic lymphadenectomy, with special histology sub-types (other than squamous cell carcinoma—SCC, adenocarcinoma or adenosquamous carcinoma), allergy to iodine or patients with SLN analyzed with standard H&E only, were excluded.

Surgical approach was performed by minimally invasive surgery (laparoscopic or robotic) at surgeon’s discretion until October 2018 (Ramirez 2018) and for tumors < 2 cm in compliance with European Society of Gynaecological Oncology (ESGO) statement and National Comprehensive Cancer Network (NCCN) guidelines, using protecting maneuvers (no uterine manipulator and vaginal cuff formation in patients with tumor completely excised at pre-operative conization), after that date (Koh 2019; Querleu 2020). Laparotomy approach was performed at surgeon’s discretion until October 2018 (Ramirez 2018) and for tumors ≥ 2 cm after that date. Uterine surgery involved radical hysterectomy in patients who did not wish to retain fertility; and cervical conization or cervical biopsy followed by NACT [as part of a previously described clinical trial, NCT02323841—in press Fertility and Sterility (ClinicalTrials.gov Identifier: NCT02323841) (Clinicaltrial 2014)], in patients who desired to preserve fertility with tumor diameter ≤ 2 cm and > 2 cm, respectively. Patients with clinical diagnosis of FIGO stage IIB, underwent NACT followed by radical hysterectomy. All patients undergoing open surgery were treated with radical hysterectomy.

FIGO stage refers to pathologic stage for patients who underwent primary surgery and to clinical stage at diagnosis for patients undergoing NACT.

### Sentinel lymph node assessment

Technique and method for SLN mapping was the same in all cases. SLN was detected after 1 ml superficial and deep cervical injections of ICG (diluted with sterile water at 1.25 mg/ml) at 3 and 9 o’clock. About 15 min after cervical injection, pelvic retroperitoneal space was opened, and lymph nodes were assessed with near infra-red (NIR) camera: Visera Elite II (Olympus, Tokyo, Japan) and Pinpoint (Stryker, Kalamazoo, Michigan, US) in case of laparoscopic or Da Vinci Xi (Intuitive, Sunnyvale, California, US) in case of robotic approach; SLN in open surgery was detected with SPY Portable Handheld Imager (SPY-PHI) camber (Stryker, Kalamazoo, Michigan, US). SLN was defined as the ICG-positive pelvic node closest to the tumor; retroperitoneal spaces were explored with the following order to assess SLN presence: external iliac, interiliac, obturator, common iliac, parametrial and pre-sacral and low para-aortic area (Marnitz 2006; Balaya 2019). All mapped SLNs were removed and analyzed with ultrastaging (Cibula 2012) or with one-step nucleic acid amplification (OSNA) (Bizzarri 2020).

Ultrastaging procedure was performed as follows: hematoxylin and Eosin (H&E) negative nodes were entirely sectioned at 150 μm intervals, until exhaustion of the lymph node, according to the length of the long axis. Each level was examined by H&E and AE1/AE3. Low volume disease was defined by the American Joint Committee on Cancer (AJCC) (Schwartz 2002): macro-metastases were defined as cancer deposits larger than 2.0 mm; micro-metastases were defined as deposits between 0.2 and 2.0 mm; and isolated tumor cells were defined as deposits no greater than 0.2 mm, including the presence of single non-cohesive cytokeratin-positive cancer cells.

OSNA analysis of SLNs was performed as per previously described protocol.

Systematic bilateral pelvic lymphadenectomy was performed in all patients, regardless SLN mapping. Non-SLNs were examined with routine H&E staining.

### Statistical analysis

Standard descriptive statistics were used to evaluate the distribution of each variable. Continuous variables were reported as median and categorical variables as frequency and percentage. Comparison of each variable between the groups of patients have been performed with *t* test for continuous variables and Chi-square or Fisher’s exact test for categorical variables. Sensitivity, negative predictive value, and false-negative rates were calculated per patient and per hemi-pelvis. We considered findings to be false negative when lymphatic mapping showed drainage to one or more SLNs in a hemi-pelvis, biopsy of the SLN(s) revealed no metastases, and the patient had at least one metastatic non-SLN. A positive non-SLN in a hemi-pelvis with no SLN identified on lymphatic mapping was not considered to indicate a false-negative finding as our practice was to perform a complete pelvic lymphadenectomy in any case. All *p* values reported were two sided and a *p* value < 0.05 was considered statistically significant. Analysis was computed using SPSS version 26.0 (IBM Corporation 2018, Armonk, NY: IBM Corp.).

## Results

### Entire cohort characteristics

Eighty-five patients met inclusion criteria and underwent ICG injection to detect SLN. Twenty-seven (31.8%) underwent open and 58 (68.2%) underwent minimally invasive SLN mapping. Of these, 43 (50.6%) underwent laparoscopic and 15 (17.6%) robotic approach.

Table 1 shows characteristics of the entire cohort. Nine (10.6%) patients were submitted to NACT before radical hysterectomy. Most of patients had SCC histology (56.5%), grade 2 (51.8%), maximum tumor diameter < 4 cm (56.5%) and underwent type B or C radical hysterectomy (82.4%). Overall, 63 (74.1%) patients had bilateral SLN mapping, while 15 (17.6%) and 7 (8.2%) had unilateral and no mapping, respectively. One-hundred and seventy-six SLNs were detected and retrieved. Median number of SLNs removed was 2 (range, 1–5) per patient. The most frequent site of SLN mapping was external iliac in 76 (58.5%), followed by obturator in 38 (29.2%), internal iliac in 7 (5.4%) and common iliac in 5 (3.8%) cases. Two (1.5%) and two (1.5%) patients had para-aortic and pre-sacral mapping, respectively, but none of these was isolated. No patient presented allergic reaction to ICG in the entire cohort.

**Table 1:** Patients’ characteristics

Characteristic	*N* = 85, median (range, %)
Age (years)	44 (27–80)
BMI (kg/m2)	23.0 (17.3–36.0)
Neo-adjuvant chemotherapy	
No	76 (89.4)
Yes	9 (10.6)
Pre-operative conization	
No	58 (68.2)
Yes	27 (31.8)
Approach	
Laparoscopy	43 (50.6)
Robot	15 (17.6)
Laparotomy	27 (31.8)
Type of surgery on uterus/cervix at time of SLN biopsy	
Conization	7 (8.2)
Biopsy only	2 (2.4)
Type A RH	6 (7.1)
Type B RH	31 (36.5)
Type C RH	39 (45.9)
SLN mapping	
No	7 (8.2)
Unilateral	15 (17.6)
Bilateral	63 (74.1)
SLN analysis	
No mapping	7 (8.2)
Ultra staging	49 (57.6)
OSNA	29 (34.1)
Median number SLN	2 (1–5)
Histology	
SCC	48 (56.5)
Adenocarcinoma	34 (40.0)
Adenosquamous	3 (3.5)
Grade	
1	9 (10.6)
2	44 (51.8)
3	21 (87.1)
Unknown	11 (12.9)
LVSI	
Negative	45 (52.9)
Positive	35 (41.2)
Unknown	5 (5.9)
Depth of stromal infiltration	5 (0.5–25)
Maximum tumor diameter (mm)	20 (1–65)
Tumor diameter	
≤ 20 mm	45 (52.9)
> 20 mm	40 (47.1)
FIGO stage 2018	
IA1	7 (8.2)
IA2	4 (4.7)
IB1	19 (22.4)
IB2	20 (23.5)
IB3	6 (7.1)
IIA1	4 (4.7)
IIB	5 (5.9)
IIIC1p	20 (23.5)
Number or removed lymph nodes	18 (5–86)
SLN largest dimension metastasis	
No mapping	7 (8.2)
No	60 (70.6)
ITC	3 (3.5)
Micro	13 (15.3)
Macro	2 (2.4)

*BMI* body mass index, *SLN* sentinel lymph node, *OSNA* one-step nucleic acid amplification, *SCC* squamous cell carcinoma, *LVSI* lymph-vascular space involvement, *FIGO* international federation of gynecology and obstetrics

Overall, 20 (23.5%) patients had metastatic lymph nodes, and 18 (21.2%) had metastatic SLN. Of these, 3 (3.5%) had ITCs, 13 (15.3%) had micro- and 2 (2.4%) had macro-metastases.

In the entire cohort, when calculated per-patient, the sensitivity of SLN biopsy was 90.0% (95% CI 68.3–98.8%) and the negative predictive value was 96.7% (95% CI 88.6–99.1%). When calculated per hemi-pelvis, the sensitivity of SLN biopsy was 97.1% (95% CI 85.1–99.9%) and the negative predictive value was 99.1% (95% CI 93.9–99.9%) (Supplementary Table 1).

### Comparison of open and minimally invasive approach

**Fig. 1:**
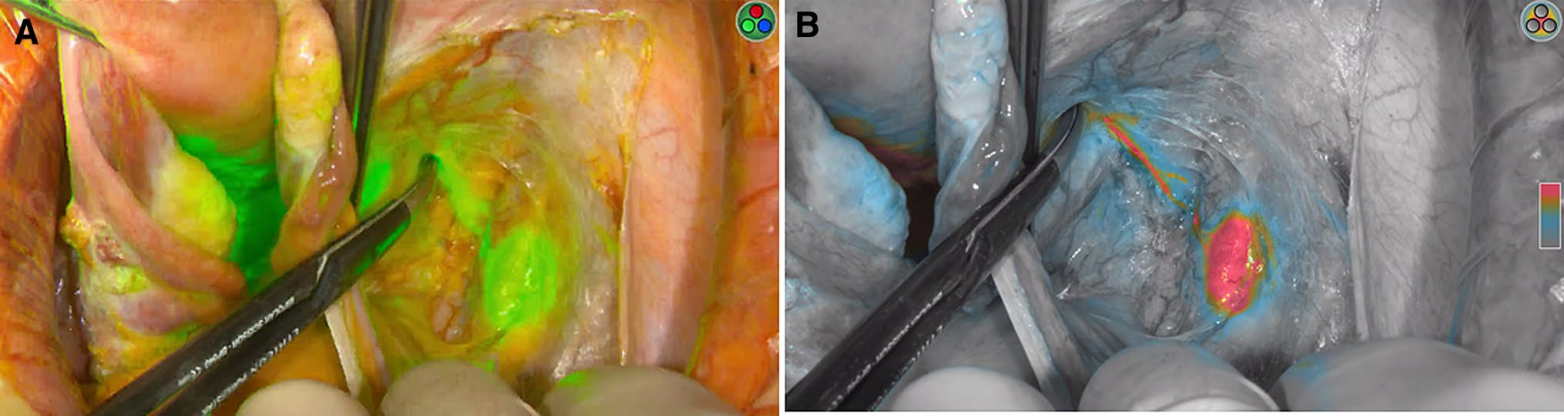
Right external iliac SLN detected with ICG green mode (1**a**) and color-segmented fluorescence (1**b**) during open surgery

**Fig. 2:**
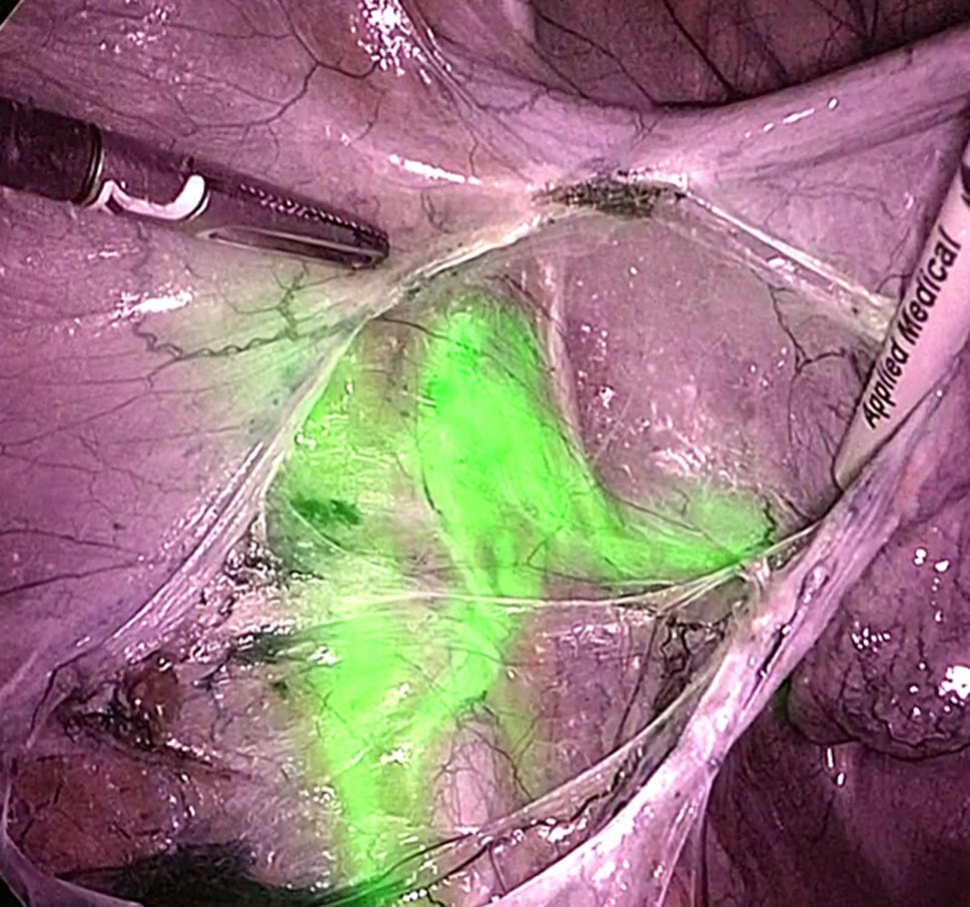
Left external iliac SLN detected with ICG during laparoscopic surgery

Comparison of characteristics of minimally invasive (58, 68.2%) and open approach (27, 31.8%) is reported in Table 2. Patients who underwent open approach reported higher rate of grade 3 (*p* = 0.028) and positive LVSI (*p* = 0.003), deeper stromal infiltration (median 8 mm vs. 5 mm, *p* = 0.016), larger maximum tumor diameter (median 25 mm vs. 15 mm, *p* = 0.003) and higher FIGO stage (*p* = 0.039). Despite these differences, no difference in any SLN mapping or in SLN bilateral detection was evident between open and minimally invasive approach (*p* = 0.850 and *p* = 0.222, respectively). Moreover, there was no difference in median number of SLNs mapped and retrieved between the two approaches (2 in both groups, *p* = 0.165) and in site of SLN mapping per hemi-pelvis (right side, *p* = 0273 and left side, *p* = 0.618). Figures 1 and 2 show two examples of SLN mapping in open and minimally invasive surgery, respectively.

**Table 2:** Comparison of characteristics of patients undergoing SLN mapping by laparotomy (LPT) and minimally invasive surgery (MIS)

Characteristic	LPT = 27, %	MIS = 58, %	*p* value
Age (years)	45 (27–80)	34.5 (29–77)	0.421
BMI (kg/m2)	23.0 (18.7–35.0)	23.3 (17.3–36.0)	0.718
Neo-adjuvant chemotherapy			
No	23 (85.2)	53 (91.4)	0.456
Yes	4 (14.8)	5 (8.6)	
Histology			0.483
SCC	16 (59.3)	32 (55.2)	
Adenocarcinoma	11 (40.7)	23 (39.7)	
Adenosquamous	0	3 (5.2)	
Grade^a^			**0.028**
1	2 (7.4)	7 (12.1)	
2	11 (40.7)	33 (56.9)	
3	12 (44.4)	9 (15.5)	
Unknown	2 (7.4)	9 (15.5)	
LVSI^a^			**0.003**
Negative	8 (29.6)	37 (63.8)	
Positive	17 (63.0)	18 (31.0)	
Unknown	2 (7.4)	3 (5.2)	
Depth of stromal infiltration	8 (0.4–25)	5 (0.6–17)	**0.016**
Maximum tumor diameter (mm)	25 (5–65)	15 (1–50)	**0.003**
Tumor diameter			**0.005**
≤ 20 mm	8 (29.6)	37 (63.8)	
> 20 mm	19 (70.4)	21 (36.2)	
FIGO stage 2018			**0.039**
IA1	0	7 (12.1)	
IA2	0	4 (6.9)	
IB1	5 (18.5)	14 (24.1)	
IB2	7 (25.9)	13 (22.4)	
IB3	5 (18.5)	1 (1.7)	
IIA1	1 (3.7)	3 (5.2)	
IIB	3 (11.1)	2 (3.4)	
IIIC1p	6 (22.2)	14 (24.1)	
SLN mapping			0.850
No	2 (7.4)	5 (8.6)	
Yes	25 (92.6)	53 (91.4)	
SLN detection^b^			0.222
Unilateral	7 (28.0)	8 (15.1)	
Bilateral	18 (72.0)	45 (84.9)	
SLN analysis^b^			0.133
Ultrastaging	19 (76.0)	30 (56.6)	
OSNA	6 (24.0)	23 (43.4)	
Number of SLN^b^			0.476
1	5 (20.0)	7 (13.2)	
2	17 (68.0)	30 (56.6)	
3	1 (4.0)	9 (17.0)	
4	1 (4.0)	5 (9.4)	
5	1 (4.0)	1 (1.9)	
6	0	1 (1.9)	
Median number of SLN	2 (1–5)	2 (1–6)	0.165
Median number of removed lymph nodes	23 (8–57)	18 (5–86)	0.197
Site of mapping of first Right SLN^c^			0.273
Obturator	6 (26.1)	13 (31.0)	
Internal iliac	0	4 (9.5)	
External iliac	16 (69.6)	21 (50.0)	
Common iliac	0	3 (7.1)	
Para-aortic	1 (4.3)	1 (2.4)	
Site of mapping of first Left SLN^d^			0.618
Obturator	6 (30.0)	13 (31.0)	
Internal iliac	0	3 (7.1)	
External iliac	13 (65.0)	25 (59.5)	
Common iliac	1 (5.0)	1 (2.4)	
SLN metastasis^b^			0.801
No	20 (80.0)	40 (75.5)	
ITC	1 (4.0)	2 (3.8)	
Micro	4 (16.0)	9 (17.0)	
Macro	0	2 (3.8)	

Bold significance values are *p* < 0.05

*BMI* body mass index, *SCC* squamous cell carcinoma, *LVSI* lymph-vascular space involvement, *FIGO* international federation of gynecology and obstetrics, *SLN* sentinel lymph node, *OSNA* one-step nucleic acid amplification, *ITC* isolated tumor cell

^a^ Analysis performed on cases with available data only

^b^ Analysis performed on 78 cases with uni/bilateral mapping

^c^ analysis performed on 65 right hemipelvis mapping, 7 patients had pelvic mapping but site not specified; total number of SLNs retrieved: 176

^d^ analysis performed on 62 left hemipelvis mapping, 7 patients had pelvic mapping but site not specified; total number of SLNs retrieved: 176

In the sub-group of laparotomy patients, per-patient sensitivity of SLN biopsy was 83.3% (95% CI 35.9–99.6%) and negative predictive value was 95.0% (95% CI 76.0–99.1%). When calculated by hemi-pelvis, the sensitivity of SLN biopsy was 88.9% (95% CI 51.7–99.7%) and the negative predictive value was 97.1% (95% CI 84.3–99.5%) (Supplementary Table 1). For what concerns minimally invasive group, the sensitivity of SLN biopsy was 92.9% (95% CI 66.1–99.8%) and the negative predictive value was 97.5% (95% CI 85.5–99.6%) in the per-patients and they were 100.0% (95% CI 86.8–100.0%) and 100.0% respectively, in the per-hemipelvis calculation (Supplementary Table 1). No difference in per-patient sensitivity was noted between laparotomy and minimally invasive surgery (*p* = 0.300).

### Comparison of no/unilateral and bilateral SLN detection

Analysis of variables associated with bilateral SLN detection, compared with no/unilateral SLN detection within the entire cohort, is reported in Table 3. None of the analyzed characteristics was significantly different between the two groups. In particular, no surgery-related, or tumor-related variable was associated with higher bilateral SLN mapping. When we divided the study period in two parts, considering the first as “learning period” (33 patients, until 12/2018) and the second “experienced period” (52 patients, from 01/2019), no difference was observed in bilateral SLN detection rate (*p* = 0.450).

**Table 3:** Factors associated with bilateral detection in the entire cohort

Characteristic	No/unilateral mapping (*N* = 22)	Bilateral mapping (*N* = 63)	*p* value
Age (years)	48 (29–80)	42 (27–75)	0.121
BMI (kg/m2)	24.2 (18.1–33.2)	23 (17.3–36)	0.905
Histology			0.618
SCC	11 (50.0)	37 (58.7)	
Non-SCC	11 (50.0)	26 (41.3)	
Grade^a^			0.737
1	3 (13.6)	6 (9.5)	
2	11 (50.0)	33 (52.4)	
3	7 (31.8)	14 (22.2)	
Unknown	1 (4.5)	10 (15.9)	
LVSI^a^			0.444
Negative	10 (45.5)	35 (55.5)	
Positive	11 (50.0)	24 (38.1)	
Unknown	1 (4.5)	4 (6.3)	
Maximum tumor diameter (mm)	20.5 (1–57)	19 (1–65)	0.634
Tumor diameter			0.464
≤ 20 mm	10 (45.5)	35 (55.6)	
> 20 mm	12 (54.5)	28 (44.4)	
Depth of stromal infiltration	5 (0.6–19)	5 (0.4–25)	0.953
FIGO stage 2018			0.602
IA-IB	16 (72.7)	40 (63.5)	
IIA-IIB/IIIc1p	6 (27.3)	23 (36.5)	
Pathologic parametrial infiltration			0.332
No	22 (100.0)	57 (90.5)	
Yes	0	6 (9.5)	
Pathologic lymph node metastasis			0.918
No	17 (77.3)	48 (76.2)	
Yes	5 (22.7)	15 (23.8)	
Neo-adjuvant treatment			0.435
No	21 (95.5)	55 (87.3)	
Yes	1 (4.5)	8 (12.7)	
Approach			0.299
Laparotomy	9 (40.9)	18 (28.6)	
MIS	13 (59.1)	63 (71.4)	
SLN metastasis^b^			0.739
No	11 (73.3)	49 (77.8)	
Yes	4 (26.7)	14 (22.2)	
SLN metastasis^b^			0.798
No	11 (73.3)	49 (77.8)	
ITC	1 (6.7)	2 (3.2)	
Micro	3 (20.0)	10 (15.9)	
Macro	0	2 (3.2)	

*BMI* body mass index, *SCC* squamous cell carcinoma, *LVSI* lymph-vascular space involvement, *FIGO* international federation of gynecology and obstetrics *SLN* sentinel lymph node, *ITC* isolated tumor cell

^a^ Analysis performed on cases with available data only

^b^ 7 patients did not have SLN mapping therefore no information on SLN metastasis available

## Discussion

The aim of this study was to compare the bilateral SLN detection rate with ICG of patients undergoing open and minimally invasive radical hysterectomy for cervical cancer. Following the publication of the LACC trial (Ramirez 2018), which demonstrated survival advantage in patients operated with laparotomy approach, international societies have changed their recommendations in terms of surgical approach for radical hysterectomy in early-stage cervical cancer (Koh 2019, Querleu 2020). Multiple subsequent observational studies have confirmed LACC trial results, particularly for patients with tumors larger than 2 cm (Nitecki 2020, Pedone Anchora 2020). SLN has become essential part of the operative management of early-stage cervical cancer, as it provides important information on lymph nodes at highest probability of metastasis and it allows a thorough analysis of these nodes by ultrastaging (Cibula 2012) or OSNA (Bizzarri 2020). Such analysis allows to detect low-volume metastases, which represents an important information, having some authors described the negative prognostic impact of micrometastases (Kocian 2020); nevertheless, not all studies support the negative impact of micrometastases in cervical cancer (Guani 2020). Moreover, ICG has been demonstrated to be superior to other tracers alone (such as blue dye or radioactive tracers), and comparable with combination of blue and radioactive tracer in detecting SLNs in cervical cancer (Di Martino 2017). With this background, we intended to assess whether ICG provides such a high bilateral SLN detection rate in open surgery as in minimally invasive surgery, in view of the clinical practice change that many centers across the world had with regards to the surgical approach.

With our results, we showed no difference in any SLN and bilateral SLN detection between patients undergoing SLN mapping with open surgery compared to laparoscopic or robotic surgery. Moreover, with an overall and bilateral detection rate of 92.6% and 72.0%, SLN mapping with ICG in laparotomy is in line with previously reported pooled data from a diagnostic review, showing overall and bilateral detection rate of 91% and 60%, respectively (Tax 2015).

Differences in tumor characteristics between patients undergoing open and minimally invasive approach is likely to be related to the approach selection. Even though not significant, the difference in bilateral detection rate between the two approaches (72.0% in open versus 84.9% in minimally invasive), could be explained by the tumor characteristics of patients undergoing open approach. In fact, as previously described by (Dostálek 2018), bilateral SLN detection in large volume cervical cancer was lower, compared to smaller tumors.

Regarding sensitivity and negative predictive value in the per-patient analysis, our results demonstrated that there was no significant difference between patients undergoing open ICG SLN (83.3–95.0%, respectively) and minimally invasive ICG SLN (92.9–97.5%, respectively). The same applies for the per-hemipelvis analysis. When compared with other literature studies, we found sensitivity from open ICG SLN in the present series to be slightly lower compared with other sensitivities reported in the literature (ranging from 91.0 to 96.4%), with a negative predictive value concordant with other series (ranging from 91.0 to 100%) (Cibula 2012; Tax 2015; Salvo 2017).

This is not the first study reporting the use of ICG to detect SLN in open surgery for cervical cancer. However, to date, only few case reports or small series were reported (Furukawa 2010; Crane 2011; van der Vorst 2011; Schaafsma 2012; Buda 2016; Rychlik 2016; Snyman 2018; Bedyńska 2019). Table 4 shows the characteristics of the studies which reported on the use of ICG to detect SLN in laparotomy. On the other hand, SLN in open surgery has been largely described with the use of other tracers such as blue dye, radioactive tracer (technetium) or combined technique (Silva 2005; Wydra 2006; Altgassen 2007; Kara 2008; Dostalek 2020). Nevertheless, ICG shows some advantages compared to other tracers: the low-risk of allergic reaction compared to blue dye (Papadia 2017); possibility of performing SLN biopsy in one-step at the time of surgery and the absence of patients exposure to ionizing radiation compared to radioactive tracer; lastly, it allows a real-time visualization of lymphatic channels.

**Table 4:** Literature review showing previous cases of SLN detected with ICG in open surgery for cervical cancer

Authors	Year	Total number of cervical cancer cases	Number of open cases with ICG	Tracer to detect SLN	Setting	Overall detection rate	Bilateral detection rate	SLN metastasis	Sensitivity	NPV
Crane et al.	2010	10	10	Methylene blue + ICG	Primary surgery	60%	30%	11%	100%	100%
Furukawa et al.	2010	12	12	ICG	Primary surgery	83%	83%	16.7%	100%	100%
Van der Vorst et al.	2011	9	9	ICG	Primary surgery: 8 No uterine surgery: 1	100%	88.9%	22.2%	100%	100%
Schaafsma et al.	2012	18	17	ICG	Primary surgery: 17 After NACT: 1	78%	61%	33%	83.3%	92.3%
Buda et al.	2016	2	2	ICG	Primary surgery	100%	100%	0	100%	100%
Rychlik et al.	2016	1	1	ICG	Primary surgery	100%	100%	0	100%	100%
Snyman et al.	2018	72	NS	Methylene blue + ICG	Primary surgery	87.5%^a^	30.5%^a^	NS	85.7%^a^	100%^a^
Bedyńska et al.	2019	6	NS	ICG	Primary surgery	67.0%	56.0%	0	NS	NS
Present study	2020	85	27	ICG	Primary surgery: 23 After NACT: 4	92.6%	72.0%	18.5%	83.3%	95.0%

*SLN* sentinel lymph node, *NIR* near infrared, *NPV* negative predictive value, *ICG* indocyanine green, *NS* not stated in the original article, *NACT* neo-adjuvant chemotherapy

^a^ Not only in open-ICG cases

The main limitation of the present study is the possible selection bias for patients submitted to open surgery; on the other hand, we have to acknowledge that this is the first study reporting the use of ICG in open surgery in a relatively large number of patients with cervical cancer, with a novel near infrared technology.

## Conclusion

In conclusion, the use of ICG to detect SLN in cervical cancer treated with open surgery allows a bilateral detection, sensitivity and negative predictive value comparable to minimally invasive surgery or with open surgery using other tracers. Performance of open SLN biopsy with ICG hits the standards described for cervical cancer. The advantages of ICG compared to other tracers make it a promising tool to detect SLN in an era where open surgery and SLN biopsy has become essential part of the personalized treatment for cervical cancer.

## Electronic supplementary material

Below is the link to the electronic supplementary material.

Supplementary file1 (DOCX 20 kb)

## Data Availability

All data and materials as well as software application or custom code support their published claims and comply with field standards.
