# Supplementary material for: Sentinel lymph node mapping with indocyanine green in cervical cancer patients undergoing open radical hysterectomy: a single-institution series
Source: J Cancer Res Clin Oncol. 2020 Sep 30;147(3):649–59. doi: 10.1007/s00432-020-03393-6 (PMC7873085; doi:10.1007/s00432-020-03393-6)
Supplement: Supplementary file 1 — Supplementary file1 (DOCX 20 kb) [file 432_2020_3393_MOESM1_ESM.docx]

**Supplementary Table 1.** Performance of SLN biopsy compared with pelvic lymph nodes in patients who had any mapping (patients with no SLN mapping or hemi-pelvis with no SLN mapping excluded).

| **Entire cohort** | | | | | | | | | | | | | | |
| --- | --- | --- | --- | --- | --- | --- | --- | --- | --- | --- | --- | --- | --- | --- |
| **Per patient** | | | | | | | | | | | | | | |
| **Statistic** | **Value** | **95% CI** |  |  | | **Disease** | | | | | | | |  |
| Sensitivity | 90.00% | 68.30% to 98.77% |  | **Test** | | **Present** | | **N** | | **Absent** | | **N** | | **Total** |
| Negative Likelihood Ratio | 0.10 | 0.03 to 0.37 |  | **Positive** | | True Positive | | a= 18 | | False Positive | | c= 0 | | a + c = 18 |
| Disease prevalence * | 25.64% | 16.42% to 36.79% |  | **Negative** | | False Negative | | b= 2 | | True Negative | | d= 58 | | b + d = 60 |
| Negative Predictive Value * | 96.67% | 88.62% to 99.08% |  | **Total** | |  | | a + b = 20 | |  | | c + d = 58 | | Total = 78 |
| Accuracy * | 97.44% | 91.04% to 99.69% |  |  | |  | |  | |  | |  | |  |
| **Per hemipelvis** | | | | | | | | | | | | | | |
| **Statistic** | **Value** | **95% CI** |  |  | | **Disease** | | | | | | | |  |
| Sensitivity | 97.14% | 85.08% to 99.93% |  | **Test** | | **Present** | | **N** | | **Absent** | | **N** | | **Total** |
| Negative Likelihood Ratio | 0.03 | 0.00 to 0.20 |  | **Positive** | | True Positive | | a= 34 | | False Positive | | c= 0 | | a + c = 34 |
| Disease prevalence * | 24.82% | 17.94% to 32.79% |  | **Negative** | | False Negative | | b= 1 | | True Negative | | d= 106 | | b + d = 107 |
| Negative Predictive Value * | 99.07% | 93.89% to 99.86% |  | **Total** | |  | | a + b = 35 | |  | | c + d = 106 | | Total = 141 |
| Accuracy * | 99.29% | 96.11% to 99.98% |  |  | |  | |  | |  | |  | |  |
| **Laparotomy group** | | | | | | | | | | | | | | |
| **Per patient** | | | | | | | | | | | | | | |
| **Statistic** | **Value** | **95% CI** |  |  | **Disease** | | | | | | | |  | |
| Sensitivity | 83.33% | 35.88% to 99.58% |  | **Test** | **Present** | | **N** | | **Absent** | | **N** | | **Total** | |
| Negative Likelihood Ratio | 0.17 | 0.03 to 1.00 |  | **Positive** | True Positive | | a= 5 | | False Positive | | c= 0 | | a + c = 5 | |
| Disease prevalence * | 24.00% | 9.36% to 45.13% |  | **Negative** | False Negative | | b= 1 | | True Negative | | d= 19 | | b + d = 20 | |
| Negative Predictive Value * | 95.00% | 76.05% to 99.13% |  | **Total** |  | | a + b = 6 | |  | | c + d = 19 | | Total = 25 | |
| Accuracy * | 96.00% | 79.65% to 99.90% |  |  |  | |  | |  | |  | |  | |
| **Per hemipelvis** | | | | | | | | | | | | | | |
| **Statistic** | **Value** | **95% CI** |  |  | **Disease** | | | | | | | |  | |
| Sensitivity | 88.89% | 51.75% to 99.72% |  | **Test** | **Present** | | **N** | | **Absent** | | **N** | | **Total** | |
| Negative Likelihood Ratio | 0.11 | 0.02 to 0.71 |  | **Positive** | True Positive | | a= 8 | | False Positive | | c= 0 | | a + c = 8 | |
| Disease prevalence * | 20.93% | 10.04% to 36.04% |  | **Negative** | False Negative | | b= 1 | | True Negative | | d= 34 | | b + d = 35 | |
| Negative Predictive Value * | 97.14% | 84.27% to 99.54% |  | **Total** |  | | a + b = 9 | |  | | c + d =  34 | | Total = 43 | |
| Accuracy * | 97.67% | 87.71% to 99.94% |  |  |  | |  | |  | |  | |  | |
| **Minimally-invasive surgery group** | | | | | | | | | | | | | | |
| **Per patient** | | | | | | | | | | | | | | |
| **Statistic** | **Value** | **95% CI** |  |  | **Disease** | | | | | | | |  | |
| Sensitivity | 92.86% | 66.13% to 99.82% |  | **Test** | **Present** | | **N** | | **Absent** | | **N** | | **Total** | |
| Negative Likelihood Ratio | 0.07 | 0.01 to 0.47 |  | **Positive** | True Positive | | a= 13 | | False Positive | | c= 0 | | a + c = 13 | |
| Disease prevalence * | 26.42% | 15.26% to 40.33% |  | **Negative** | False Negative | | b= 1 | | True Negative | | d= 39 | | b + d = 40 | |
| Negative Predictive Value * | 97.50% | 85.51% to 99.61% |  | **Total** |  | | a + b =  14 | |  | | c + d =  39 | | Total = 53 | |
| Accuracy * | 98.11% | 89.93% to 99.95% |  |  |  | |  | |  | |  | |  | |
| **Per hemipelvis** | | | | | | | | | | | | | | |
| **Statistic** | **Value** | **95% CI** |  |  | **Disease** | | | | | | | |  | |
| Sensitivity | 100.00% | 86.77% to 100.00% |  | **Test** | **Present** | | **N** | | **Absent** | | **N** | | **Total** | |
| Negative Likelihood Ratio | 0.00 |  |  | **Positive** | True Positive | | a= 26 | | False Positive | | c= 0 | | a + c = 26 | |
| Disease prevalence * | 26.53% | 18.12% to 36.41% |  | **Negative** | False Negative | | b= 0 | | True Negative | | d= 72 | | b + d = 72 | |
| Negative Predictive Value * | 100.00% |  |  | **Total** |  | | a + b = 26 | |  | | c + d = 72 | | Total = 98 | |
| Accuracy * | 100.00% | 96.31% to 100.00% |  |  |  | |  | |  | |  | |  | |

* These values are dependent on disease prevalence.
